# *Pseudomonas protegens* FJKB0103 Isolated from Rhizosphere Exhibits Anti-Methicillin-Resistant *Staphylococcus aureus* Activity

**DOI:** 10.3390/microorganisms10020315

**Published:** 2022-01-28

**Authors:** Hui Zhao, Lu Liu, Lingshuang Yang, Qihui Gu, Ying Li, Jumei Zhang, Shi Wu, Moutong Chen, Xinqiang Xie, Qingping Wu

**Affiliations:** Guangdong Provincial Key Laboratory of Microbial Safety and Health, State Key Laboratory of Applied Microbiology Southern China, Institute of Microbiology, Guangdong Academy of Sciences, Guangzhou 510070, China; zhaohuichinese@163.com (H.Z.); luckyliulululu@163.com (L.L.); yangls8272@163.com (L.Y.); guqh888@163.com (Q.G.); liying@gdim.cn (Y.L.); zhangjm926@126.com (J.Z.); wushiloveyou@126.com (S.W.); cmtoon@hotmail.com (M.C.)

**Keywords:** *Staphylococcus aureus*, *Pseudomonas*, DAPG, biofilm

## Abstract

*Staphylococcus aureus* is amongst the most virulent pathogens, causing chronic and life-threatening human infections. Methicillin-resistant *S. aureus* (MRSA) are multidrug-resistant strains, and the ability of forming a biofilm reduces their sensitivity to antibiotics. Thus, the alternative compounds inhibiting both resistant strains and biofilm formation are in high demand. In our study, the strain FJKB0103 was isolated from the rhizosphere of *Garcinia mangostana*, showing strong anti-MRSA activity. We performed molecular phylogenic analysis, analyzed average nucleotide identity (ANI), in silico DNA-DNA hybridization (*is*DDH), and biochemical characteristics to identify strain FJKB0103 as *Pseudomonas protegens*. Herein, the genome of strain FJKB0103 was sequenced and subjected to antiSMASH platform, mutational, and functional analyses. The FJKB0103 draft genome was 6,776,967 bp with a 63.4% G + C content, and 16 potential secondary compound biosynthetic clusters in *P. protegens* FJKB0103 were predicted. The deletion mutant and complementary analysis suggested that DAPG was the anti-MRSA compound. Further tests showed that MRSA strains were sensitive to DAPG, and the lysis of bacterial cells was observed at a high concentration of DAPG. Additionally, DAPG inhibited the biofilm formation of MRSA at subinhibitory concentration. These results suggested that DAPG might be a good alternative treatment to control infections caused by MRSA.

## 1. Introduction

*Staphylococcus aureus* is a common opportunistic Gram-positive pathogen that has ubiquitous distribution in nature, such as on the skin and mucosae of the human population, food, air, and water. *S. aureus* can cause wound infections and toxin-mediated syndromes as well as systemic and life-threatening diseases [1]. Antibiotic application is the major method to control an *S. aureus* infection, but the extensive use and misuse of antibiotics in both human and animal medicine have led to an escalating challenge with multidrug-resistant bacterial strains, especially Methicillin-resistant *S. aureus* (MRSA) [2]. MRSA was first reported in the 1960s [3], and it has been recognized as a major cause of healthcare-associated infections worldwide due to its multidrug resistance to almost all the currently available antibiotics except vancomycin and teicoplanin [4]. Furthermore, biofilm-associated bacteria show resistance to antibiotics: biofilm matrix protects the bacteria from antibiotics by limiting their diffusion or repulsion, and cells within the biofilm, particularly those deep within the matrix, exist in a slow-growing state, which can limit the efficacy of antibiotics, especially those that target active cell processes [5]. Therefore, new treatment strategies affecting both resistant strains and bacterial biofilm formation are in crucial demand [5].

*Pseudomonas* is a diverse genus of γ-proteobacteria with more than 200 validly named species exhibiting varied lifestyles in a wide range of environments, including soil, water, plant surfaces, and animals (https://lpsn.dsmz.de/genus/pseudomonas, accessed on 20 November 2021). The complex ecological environments of *Pseudomonas* spp. promote genomic diversity. The bacteria acquire and discard genomic fragments in the process of developing a genetic repertoire customized to their special niche, especially the biosynthetic gene cluster of secondary metabolites. *Pseudomonas* spp. have received much attention in recent decades due to the production of a remarkable array of medically and agriculturally important secondary metabolites, including 2,4-diacetylphloroglucinol (DAPG), nonribosomal peptide synthase (NRPS), pyrrolnitrin (PRN), and pyoluteorin (PLT), all of which have been proved to have antimicrobial activity [6]. DAPG could suppress a wide variety of plant pathogens, including bacteria, fungi, and omycetes [7]. The main antimicrobial mechanism of DAPG is an alteration of the cell membrane leading to the dissipation of the proton gradient across the biological membranes, disorganization of hyphal tips, including alteration of the plasma membrane, vacuolization and cell content disintegration, and inhibition of zoosporogenesis, motility, and germination of zoospore [8,9,10]. DAPG is a phloroglucinol derivative and is synthesized by phl biosynthetic gene cluster (BCG) that comprises nine genes such as *phlA, phlC*, *phlB*, *phlD*, *phlE*, *phlF*, *phlG*, *phlH*, and *phlI*. Four genes (*phlABCD*) are directly involved in DAPG biosynthesis [11]. Type III polyketide synthase (encoded by gene *phlD*) catalyzes the condensation of three malonyl-CoA molecules to produce phloroglucinol (PG) [12], and the multimeric acetyltransferase complex Phl(A2C2)2B4 (encoded by gene *phlACB*) mediates the acylation of PG to form monoacetylphloroglucinol (MAPG) and of MAPG to form DAPG [13]. PhlE is involved in the transport of DAPG [14]. Gene *phlF* encodes a TetR family transcriptional regulator that represses DAPG production [15]. Gene *phlG* that codes for a DAPG special hydrolase is regulated by another TetR regulatory protein PhlH [16].

*S. aureus* Sta24-1 is one of the prevalent MRSA strains in China and is resistant to more than ten antibiotics [17]. In the present work, we reported a novel isolate FJKB0103 that inhibited the growth of MRSA strain Sta24-1. The taxonomic status of strain FJKB0103 was determined by phylogenetic analyses, genomic analysis, and phenotypic characteristics. The anti-MRSA compound was identified by genome mining and genetic analysis. The data we obtained will enrich the compound database that might be used as an alternative to control infections caused by MRSA.

## 2. Materials and Methods

### 2.1. Strains and Growth Conditions

Bacterial strains and plasmids used in this study are listed in Supplementary Table S1. Strain FJKB0103 and its mutants were grown in Luria-Bertani broth (LB) or King’s B medium (Proteose peptone No. 3, 10 g; K_2_HPO_4_, 1.5 g; MgSO_4_·7H_2_O, 1.5 g; Mannitol, 10 g; KB) at 28 °C. *Escherichia coli* DH5α was grown at 37 °C in LB medium. *Staphylococcus aureus*, *Listeria monocytogene*, and *Bacillus cereus* were grown at 37 °C in LB or PDA medium. Where indicated, media was supplemented with ampicillin (50 µg/mL) or kanamycin (50 µg/mL) and/or 5-bromo-4-chloro-3-indolyl-D-galactopyranoside (X-Gal; 40 µg/mL).

### 2.2. Isolation and Assays for Antagonistic Capacity of Strain FJKB0103

The soil samples were collected from the rhizosphere of *Garcinia mangostana* in Zhangzhou, China. Five grams of soil sample suspended in 30 mL of sterile water was incubated at 28 °C with shaking at 140 rpm. After 4 h, the suspension was serially diluted with sterile water, and 100 µL of the diluted soil suspension was spread onto KB agar. The individual colonies with different colors or morphology were selected for further purification by streaking onto fresh LB agar. For antibacterial assays, 5 μL of an overnight culture of isolated strains were dropped onto the PDA plate center, and after inoculating at 28 °C for 2 days, the LB mixed with 10% saturated pathogenic bacteria was poured on the PDA plate, inoculated at 37 °C for 12 h.

### 2.3. Genome Sequencing and Annotation

The genomic DNA of FJKB0103 was extracted by using a DNA purification kit (Magen, Guangzhou, China) according to the manufacturer’s protocols. The paired-end library was used to sequence on the Illumina Miseq 550 platform. De novo genome assembly was performed using SPAdes pipeline version 3.12, and sequences shorter than 200 bp were trimmed. The draft genomes of FJKB0103 were annotated by Prokka 1.13.7, coding sequences were identified and annotated using Prodigal, rRNAs were predicted by RNAmmer, and tRNAs and noncoding RNAs were predicted using Aragorn and Infernal, respectively.

### 2.4. Identification of Strain FJKB0103 Based on Genome

The 16S rRNA gene was amplified using primers 27F and 1492R (Table S2). The result sequence was deposited to the EzBioCloud database (https://www.ezbiocloud.net, accessed on 25 October 2021) for preliminary identification based on the sequence similarity values between strain FJKB0103 and the related species. The genes 16S rRNA, *gyrB*, *rpoB*, and *rpoD* of *Pseudomonas* species closely related to strain FJKB0103 based on 16S rDNA similarities were retrieved from their GenBank depositions or their whole genome sequences. The multiple sequence alignments were made by using CLUSTAL_W software. Phylogenetic trees were reconstructed using both neighbor-joining and the maximum likelihood methods by Mega 5 [18]. Bootstrap analysis was performed using 1000 replications. Average nucleotide identity (ANI) values were determined by using the Orthologous Average Nucleotide Identity Tool (OAT) (http://www.ezbiocloud.net/sw/oat, accessed on 28 October 2021) [19], and the two ANI scores, ANI algorithm using blast (ANIb), and ANI of orthologous genes (OrthoANI) were calculated between strain FJKB0103 and the type strains of the closely related *Pseudomonas* species. The Genome-to-Genome Distance Calculator (GGDC) was employed for in silico DNA-DNA hybridization (*is*DDH) analysis, and all three equations in the GGDC program were used (version 2.1; http://ggdc.dsmz.de/distcalc2.php, accessed on 29 October 2021) [20].

### 2.5. Genome Analysis of Putative Secondary Metabolite Clusters

The Antibiotics and Secondary Metabolite Analysis Shell pipeline (antiSMASH) was used to analyze the secondary metabolite gene clusters of strain FJKB0103 [21]. Briefly, the detection strictness was “relaxed”, and all “Extra Features”, including KnownClusterBlast, ClusterBlast, SubClusterBlast, Cluster Pfam analysis, Pfam-based GO term annotation, and ActiveSiteFinder, were employed for biosynthetic gene cluster (BGC) border prediction and analysis.

### 2.6. Construction of P. protegens FJKB0103 Deleted Mutants and Complementary Strain

To confirm the effect of predicted gene clusters on antibiotic activity, the gene clusters, of which the similarity was more than 90%, were selected for further study. We constructed in-frame deleted mutants of gene clusters encoding DAPG, PRN, PLT, and orfamide as in previous reports, respectively [16]. An in-frame deletion of the *phlD* gene involved in the biosynthesis of DAPG was made using a two-step homologous-recombination strategy. Briefly, the upstream and downstream fragments of *phlD* gene were PCR amplified using the primer pairs listed in Table S2. The PCR fragments were digested with restriction enzymes and then cloned into the suicide vector p2P24-Km to generate plasmid p2P24-ΔphlD. The suicide plasmid p2P24-ΔphlD was introduced into strain FJKB0103 by electroporation. Colonies that appeared on LB plates containing kanamycin were picked up and inoculated in liquid LB medium without antibiotics. Overnight cultures were then plated on LB agar supplemented with 20% sucrose to generate the mutants, and the mutants were confirmed by PCR. The same approach was used to construct in-frame deletion mutants of the *prnA*, *pltA*, and *orfA* genes, yielding the mutants ΔprnA, ΔpltA, and ΔorfA, respectively (Table S1). For complementary analysis, the DAPG biosynthetic gene cluster *phlACBD* was amplified using primers phl-2425-HindIII and phl-7049-KpnI and inserted into the shuttle vector pRK415, yielding the complementary plasmid pRK415-phl. The resultant plasmid was introduced into *phlD* gene deletion mutant to generate complementary strain ΔphlD-C. Mutants and complementary strains were tested for their abilities to inhibit *S. aureus*.

### 2.7. Extraction and Detection of DAPG

The extraction and detection of DAPG were performed according to a previous method [22]. *P. protegens* FJKB0103 and its mutants were grown in KB broth for 48 h at 28 °C. Samples (800 μL) were acidified with 100 μL of 10% trifluoroacetic acid (TFA) and extracted with 900 μL of ethyl acetate. The organic phase containing DAPG was evaporated to dryness and suspended in 50 μL methanol. Volumes of the extracts of 10 μL were detected by an Agilent 1260 system equipped with a C18 column (4.6 × 150 mm). The samples were eluted from the column using a gradient of 55% acetonitrile: 45% water containing 0.1% phosphoric acid over 8 min at a flow rate of 1.0 mL/min. DAPG was monitored at 270 nm.

### 2.8. Determination of the Minimum Inhibitory Concentration (MIC) and Minimum Bactericidal Concentration (MBC) of DAPG, MAPG, PG, or Vancomycin on S. aureus

The values of MIC were evaluated following the CLSI guideline: suspension of *S. aureus* strain with 0.5 McFarland was prepared by making a direct broth suspension of isolated colonies selected from a 24 h agar plate; the suspension was diluted as 1:300 into 2 mL of cation-adjusted Muller Hinton broth (CAMHB), of which the final inoculum was approximate to 5 × 10^5^ CFU/mL; 10 μL DAPG, MAPG, PG, and vancomycin (various concentrations) were added to a tube, respectively; the MIC was the concentration of the drug that resulted in no visible bacterial growth after 20 h incubation at 37 °C in an ambient air incubator [23]. Content of the tube with no visible growth was spread on LB agar and incubated for 24 h at 37 °C. LB agar with the lowest concentration and no bacterial growth was scored as MBC [24].

### 2.9. Bacteriolytic Assay

The bacteriolytic activity of DAPG to MRSA bacterial cells was evaluated as in a previous report [25]. Briefly, the *S. aureus* Sta24-1 strain was grown in tryptic soy broth (TSB) overnight at 37 °C, 180 rpm. The overnight culture of *S. aureus* Sta24-1 was diluted as 1:100 into 2 mL of TSB. *S. aureus* Sta24-1 was exposed to DAPG with different concentrations at the early stage of exponential growth, and the negative control (CK) was exposed to methanol. The optical density (OD_600_) was measured at 0, 6, 18, and 24 h after incubation with DAPG.

### 2.10. Biofilm Formation and Swarming Motility

The effect of subinhibitory levels of DAPG on *S. aureus* biofilm formation were detected as in a previous study. *S. aureus* culture was diluted as 1:100 into 3 mL TSB glass tubes, and 3 μL of DAPG at subinhibitory level was added and incubated at 37 °C stationary. After 24 h of incubation, the medium was removed by pipetting, and tubes were gently washed with sterile water, biofilms on the inner surface of glass tubes were stained with 0.1% crystal violet and quantified at 570 nm [26]. The swarming motility of *P. protegens* FJKB0103 and its mutants was studied by spotting 5 μL of an overnight cell suspension on soft (0.3% *w*/*v*) LB agar and evaluating surface swarming motility after incubation at 28 °C for 24 h [27].

## 3. Results

### 3.1. Antibiotic Activity of Isolates from Rhizosphere

Thirty-seven bacterial strains were isolated from the rhizosphere of *Garcinia mangostana*. All isolates were tested for their antibiotic activities against methicillin-resistant *Staphylococcus aureus* (MRSA) Sta24-1. A total of five isolates showed antibacterial ability against MRSA Sta24-1, including the strain FJKB0103, whose anti-MRSA activity was the best (Figure S1). Moreover, the strain FJKB0103 exhibited broad-spectrum antibiotic activities against food-borne pathogens like *Listeria monocytogenes* and *Bacillus cereus*, but not *Escherichia coli* (Figure S1).

### 3.2. General Genome Characteristics of Strain FJKB0103

The size of strain FJKB0103 draft genome was 6,776,967 bp with a G + C content of 63.4%. It consisted of 40 contigs. A total of 6170 genes were predicted, which included 6096 coding sequences (CDSs) and 74 RNA genes including 7 rRNA genes, 66 tRNAs genes, and one tmRNA gene. The general features of the strain FJKB0103 genome are summarized in Table 1.

### 3.3. Identification of Strain FJKB0103 Based on Polyphasic Taxonomy

**Figure 1:**
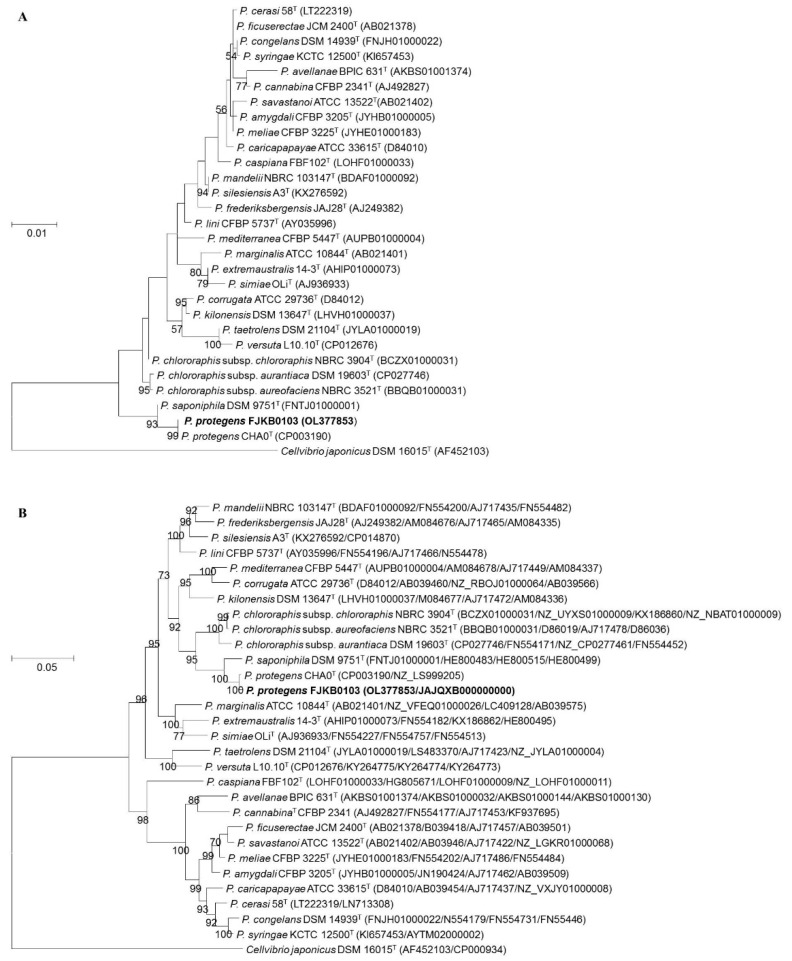
(**A**) Maximumlikelihood phylogenetic tree based on 16S rRNA gene sequences showing the relationship of strain FJKB0103 to the closely related strains. GenBank accession numbers of 16S rRNA genes are given in parentheses. (**B**) Maximum likelihood phylogenetic tree based on MLSA (16S rRNA, *gyrB*, *rpoD*, and *rpoB*) showing the relationship of strain FJKB0103 to the closely related strains. The sequences of their genes 16S rRNA (1377 bp), *gyrB* (734 bp), *rpoB* (915 bp) and *rpoD* (723 bp) were retrieved from their GenBank depositions or from their whole-genome sequences. GenBank accession numbers are given in parentheses (accession numbers are given in parentheses in the following order: 16S rRNA, *gyrB*, *rpoB*, and *rpoD* genes. *Cellvibrio japonicas* DSM 16015 ^T^ was used as an outgroup. Numbers at nodes are bootstrap values (≥50%) from 1000 repetitions. The strain FJKB0103 was highlighted in bold. ^T^ means type strain.

The 16S rRNA gene sequence of strain FJKB0103 showed the highest similarities to *P. protegens* CHA0 ^T^ and *P. saponiphila* DSM 9751 ^T^, which are members of the *P. fluorescens* group. A phylogenetic tree based on 16S rDNA and multilocus sequence analysis (MLSA) of the housekeeping genes (16S rRNA, *gyrB*, *rpoB* and *rpoD*) were built with 28 *Pseudomonas*-type strains, and *Cellvibrio japonicus* DSM 16015 ^T^ was selected as an outgroup (n = 30). Both the phylogenetic trees showed that strain FJKB0103 formed a monophyletic cluster with *P. protegens* subgroup strains including *P. protegens* CHA0 ^T^ and *P. saponiphila* DSM9751 ^T^, and the bootstrap values were high (Figure 1 and Figure S2). These data indicated that strain FJKB0103 belonged to the *P. protegens* subgroup within the *P. fluorescens* lineage.

To more accurately determine the phylogenetic position of strain FJKB0103, ANI and *is*DDH analysis were performed based on genomic sequence. The ANI values between FJKB0103 and *P. protegens* strains that included type strain *P. protegens* CHA0 ^T^ were higher than the threshold range (95–96%) for species delineation (Table 2). Furthermore, three *is*DDH values were also higher than 70% cut-off within these species. Both ANI and *is*DDH scores were lower than the values for species delineation between FJKB0103 and other *Pseudomonas* type strains (Table 2). The high similarity between FJKB0103 and CHA0 ^T^ was also confirmed at the biochemical level using API 50CH (bioMérieux, France; Table S3). All these data demonstrated that the strain FJKB0103 belonged to *P. protegens*.

### 3.4. Genome Mining to Reveal the Putative Secondary Metabolite Clusters

To predict putative secondary metabolites, such as antibiotics, bacteriocin, and NRPS genes, the genome sequence was analyzed using antiSMASH platform version 5.0. Sixteen secondary compound biosynthetic gene clusters (BGCs) were predicted in *P. protegens* FJKB0103 (Table 3 and Table S4), including DAPG, PRN, PLT, and NRPS, which showed antibiotic activity in the previous study [6]. To uncover the specific secondary metabolites, the secondary compound BGCs of several genome complete *P. protegens* strains Cab57, CHA0 ^T^, H78, SN15-2, FDAARG, and Pf-5, and six closely related type strains *P. saponiphila* DSM9751 ^T^, *P. cerasi* 58 ^T^. *P. meliae* CFBP3225 ^T^, *P. congelans* DSM14939 ^T^, *P. tremae* ICMP9151 ^T^, and *P. caspiana* FBF102 ^T^ were predicted and compared with FJKB0103 secondary compound BGCs. Strain FJKB0103 had the largest BGC numbers, while *P. caspiana* FBF102 ^T^ had the fewest (Table 3). NRPSs production was the only common feature presented in all strains, whereas DAPG, PRN, PLT, and orfamide BGCs were conserved in all the *P. protegens* strains (Table 3). The results indicated that DAPG, PRN, PLT, and orfamide might be the specific compounds for *P. protegens* species, which might be involved in the antibiotic activity of *P. protegens* FJKB0103.

### 3.5. DAPG as the Anti-MRSA Compound in P. protegens FJKB0103

**Figure 2:**
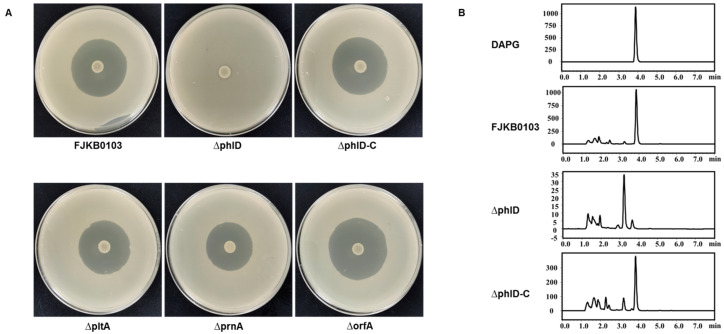
(**A**) Antimicrobial activity of *P. protegens* FJKB0103 and its mutants. For antibacterial assays, 5 μL of overnight culture of *P. protegens* FJKB0103 and its mutants were dropped onto the PDA plates center. After inoculating at 28 °C for 2 days, the LB mixed with 10% saturated *S. aureus* Sta24-1 was poured on the PDA plates, inoculated at 37 °C for 12 h. (**B**) HPLC analysis of 2,4-DAPG production of *P. protegens* FJKB0103 and its mutants in KB medium. FJKB0103, the wild-type strain of *P. protegens* FJKB0103; ∆phlD, the *phlD* gene deleted mutant of *P. protegens* FJKB0103; ∆phlD-C, the complementary strain of ∆phlD with pRK-phlACBD.

**Figure 3:**
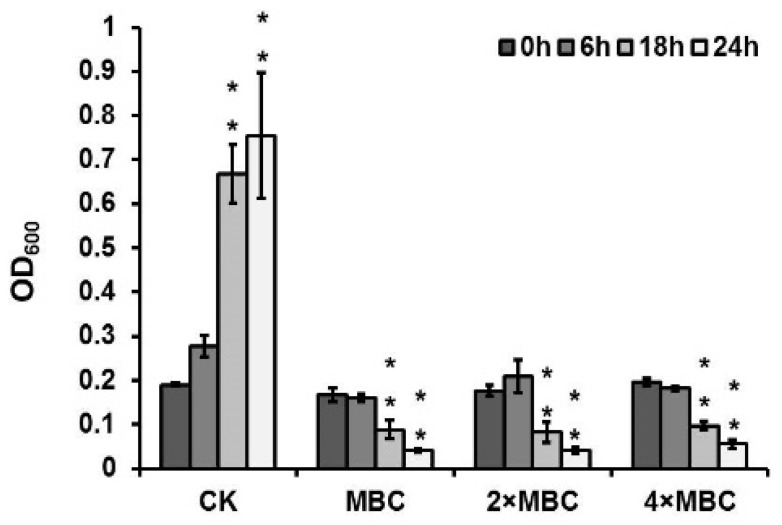
Bacteriolytic activity of DAPG against *S. aureus* Sta24-1 at 1 × MBC (32 μg/mL), 2 × MBC (64 μg/mL) and 4 × MBC (128 μg/mL). The *S. aureus* Sta24-1 was exposed to DAPG with different concentrations at the early stage of exponential growth, and the negative control (CK) was exposed to methanol. The optical density (OD_600_) was measured at 0, 6, 18, and 24 h after incubation with DAPG. Mean values of three replicates are given, and error bars indicate standard error. ** indicates *p* < 0.01.

To confirm the roles of several compounds, including DAPG, PRN, PLT, and orfamide in antibiotic activities, the genes *phlD*, *prnA*, *pltA*, and *orfA* deleted mutants were constructed, and their anti-MRSA activities were tested. The mutant ∆phlD failed to inhibit MRSA growth, and the other mutants ∆prnA, ∆pltA, and ∆orfA had the same antibacterial activities as the wild-type strain FJKB0103 (Figure 2). To further evaluate the role of gene *phlD* on antibacterial ability and DAPG production, the ∆phlD complementary strain ∆phlD-C was constructed. The anti-MRSA ability of strain ∆phlD-C was restored to the same level as wild-type strain FJKB0103, and also DAPG production was restored (Figure 2), these data supported that the DAPG biosynthetic gene cluster was involved in anti-MRSA activity of *P. protegens* FJKB0103. The anti-MRSA activity of DAPG biosynthetic gene cluster products, including DAPG, MAPG, and PG, was confirmed by detecting the MIC values. The MIC values of DAPG of all *S. aureus* strains were lower than 10 μg/μL, which were similar to those of vancomycin, but the MIC of both MAPG and PG were higher than 128 μg/μL (Table 4). Moreover, after 18 h, the bacterial suspension decreased that deal with DAPG of the MBC, 2MBC, and 4MBC level (Figure 3), indicating that the bacterial cells were lysed. All these data showed that DAPG was the anti-MRSA compound in *P. protegens* FJKB0103.

### 3.6. DAPG Affected the Biofilm Formation of S. aureus

**Figure 4:**
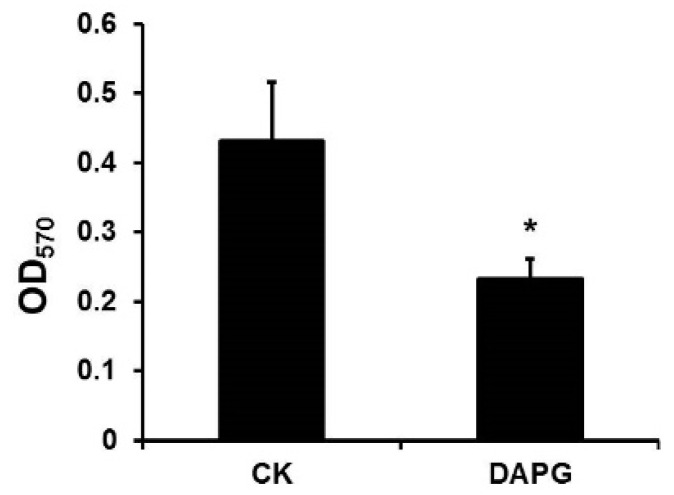
DAPG inhibited the biofilm formation of *S. aureus* Sta24-1. *S. aureus* Sta24-1 was incubated in 3 mL TSB glass tubes with 0.125 μg/mL DAPG or methanol (CK). Biofilms were stained with 0.1% crystal violet and quantified at 570 nm. Mean values of three replicates are given, and error bars indicate standard error. * indicates *p* < 0.05.

Knowing that DAPG inhibits the biofilm formation of *Bacillus subtilis* at the subinhibitory level [28], we wanted to ask if DAPG affected the biofilm formation of *S. aureus*. As biofilm formation was regulated by QS, which was related to bacterial growth [29], the subinhibitory level that did not affect the *S. aureus* strain growth was selected for detecting the effect on biofilm formation. At a subinhibitory level of DAPG, the biofilm formation of *S. aureus* Sta24-1 was decreased compared with that without DAPG (Figure 4 and Figure S3), which was consistent with data from previous studies [9,28]. These data supported the idea that DAPG affected *S. aureus* strains by both antibiotic ability and repression of biofilm formation.

## 4. Discussion

*Pseudomonas* species produce diverse secondary metabolites that have antibiotic ability. For example, DAPG, PLT, PRN, and NRPS belong to the broad-spectrum antibiotics that inhibit both bacteria and fungi, and mupirocin is used to control *S. aureus*, particularly methicillin-resistant *S. aureus* (MRSA), when other antibiotics are ineffective [30]. Phenazine-1-carboxamide and 1-acetyl-beta-carboline produced by *Pseudomonas* species also have an inhibitory effect against MRSA [31,32]. The strain FJKB0103 has 16 metabolite biosynthetic gene clusters, including DAPG and PLT, that are conversed in *P. protegens* species [26]. PG, an intermediate in DAPG biosynthesis, has a concentration-dependent influence on the expression of PLT biosynthetic genes and the production of PLT [33]. In our study, the mutant ∆phlD failed to inhibit MRSA growth completely, but the deletion of *pltA* involved in PLT biosynthesis did not affect the anti-MRSA ability, indicating that the DAPG biosynthetic gene cluster participates in the anti-MRSA ability of *P. protegens* FJKB0103. Furthermore, the MRSA strains were resistant to MAPG and PG, but sensitive to DAPG, which was in correspondence with previous reports that DAPG is more active than MAPG and PG [34]. Having said all above, DAPG is the anti-MRSA compound in *P. protegens* FJKB0103.

*Pseudomonas* species could appear in different environments, including the soil, plant surfaces, and insects, and acquire more than one gene cluster conferring antibiotic biosynthesis during the evolutionary progress. The several antibiotics produced by one strain might be beneficial to its competition with certain competitors and predators or occupation of specific habitats [6]. For example, nunamycin plays the role primarily in *Rhizoctonia solani* AG3 mycelial growth inhibition, whereas nunapeptin was essential for *Pythium aphanidermatum* inhibition in *P. fluorescens* In5 [35], and cyclic lipodepsipeptides WAP-8294A is the antibacterial compound, while HSAF (dihydromaltophilin) inhibits the fungal growth in *Lysobacter enzymogenes* OH11 [36]. *P. protegens* FJKB0103 has 16 BGCs (Table 3 and Table S4), which might be related to its special environmental niche [37]. DAPG produced by *P. protegens* FJKB0103 was the major antibacterial and antifungal compound (Figure 2 and Figure S4), and orfamide participated in the swarming motility of *P. protegens* FJKB0103 (Figure S5), however, the roles of most of the multiple secondary metabolites with diverse chemical properties produced by *P. protegens* FJKB0103 remain unclear, which will be subject to future study.

Antibiotics play a significant role in biofilm formation, and most antibiotics tend to stimulate biofilm formation at subinhibitory concentrations. For example, subinhibitory concentrations of aminoglycoside antibiotics induce biofilm formation in *P. aeruginosa* and *Escherichia coli* [38]. DAPG stimulates biofilm formation in *Azospirillum brasilense* Sp245-Rif at a subinhibitory concentration [39], whereas polymyxin B had no effect on biofilm formation of *P. aeruginosa* and *Escherichia coli* [38]. By contrast, the biofilm formation was reduced when DAPG at a subinhibitory concentration was applied to *Bacillus subtilis* NCIB3610 and MRSA (Figure 3) [28]. Antibiotics might act as signal molecules at subinhibitory concentrations, and the effect on biofilm formation can differ between antibiotic types and the strains [40].

DAPG might be a good alternative for controlling infections caused by MRSA: DAPG could lyse the bacteria and inhibit the growth at high concentrations, and represses biofilm formation at low concentrations [9,25,41,42]. Additionally, the MIC values of DAPG are similar to those of vancomycin (Table 4), which is the common antibiotic used to control MRSA infections. Furthermore, DAPG was not acutely toxic to mice when administered orally at single doses of up to 100 mg/kg [41], but the toxicity of DAPG by injection, absorbability in the digestive tract, and other pharmacodynamic properties remain unknown, thus there is more work needed for its application.

## Figures and Tables

**Table 1:** Genome statistics for *P. protegens* FJKB0103.

Features	Chromosome
Number of contigs Size (bp)	40 6,776,967
G + C content	63.4%
Number of genes	6170
Number of CDSs	6096
Number of rRNAs	7
Number of tRNAs	66
Number of tmRNAs	1

**Table 2:** Average nucleotide identity (ANI) and in silico DNA-DNA hybridization (*is*DDH) values between the genome sequence of strain FJKB0103 and closely related *Pseudomonas* species.

Species	ANIb (%) ^a^		*is*DDH (%) ^b^		
	ANIb	OrthoANI	Formula 1	Formula 2	Formula 3
*P. protegens* Cab57 (AP014522.1)	98.44	98.51	96.80	86.90	97.10
*P. protegens* CHA0 ^T^ (LS999205.1)	98.13	98.27	95.10	84.00	95.60
*P. protegens* H78 (CP013184.1)	98.09	98.21	94.00	83.70	94.70
*P. protegens* SN15-2 (CP043179.1)	98.01	98.09	92.10	83.00	93.10
*P. protegens* FDAARGOS_307 (CP022097.2)	98.00	98.11	92.10	83.00	93.10
*P. protegens* Pf-5 (NC_004129.6)	97.98	98.11	92.10	83.00	93.10
*P. saponiphila* DSM 9751 ^T^ (FNTJ00000000.1)	89.30	89.71	62.90	38.60	57.50
*P. cerasi* 58 ^T^ (LT222319.1)	77.25	78.07	19.60	22.50	19.10
*P. meliae* CFBP 3225 ^T^ (JYHE00000000.1)	77.23	77.96	19.10	22.50	18.70
*P. congelans* DSM 14939 ^T^ (NJH0000000)	77.13	78.10	19.50	22.40	19.10
*P. tremae* ICMP9151 ^T^ (LJRO00000000.1)	76.67	77.50	18.10	22.30	17.80
*P. caspiana* FBF102 ^T^ (LOHF00000000.1)	76.45	77.20	17.80	22.60	17.60

^a^ The average nucleotide identity (ANI) (ANIb and OrthoANI) was calculated using OAT software; ^b^ The *is*DDH values were calculated using the Genome-to-Genome Distance Calculator (http://ggdc.dsmz.de/distcalc2.php, accessed on 29 October 2021); ^T^ means type strain.

**Table 3:** Secondary metabolite and antibiotic gene clusters in *P.* *protegens* FJKB0103 and related strains predicted by antiSMASH ^a^.

Strains	Total	DAPG	PLT	PRN	NRPS	Bacteriocin	Others
*P. protegens* FJKB0103	16	1	1	1	7	2	4
*P. protegens* Cab57	14	1	1	1	5	2	4
*P. protegens* CHA0 ^T^	14	1	1	1	5	3	3
*P. protegens* H78	15	1	1	1	5	3	4
*P. protegens* SN15-2	16	1	1	1	6	2	5
*P. protegens* FDAARG	15	1	1	1	5	3	4
*P. protegens* Pf-5	15	1	1	1	5	3	4
*P. saponiphila* DSM 9751 ^T^	14	1	0	0	4	3	6
*P. cerasi* 58 ^T^	9	0	0	0	5	1	3
*P. meliae* CFBP 3225 ^T^	9	0	0	0	5	0	4
*P. congelans* DSM 14939 ^T^	9	0	0	0	7	0	2
*P. tremae* ICMP9151 ^T^	15	0	0	0	9	0	6
*P. caspiana* FBF102 ^T^	6	0	0	0	2	2	2

^a^ Clusters identified by antiSMASH 5.0 using the “Extra Features” settings; Abbreviations are as follows: 2,4-diacetylphloroglucinol, DAPG; pyrrolnitrin, PRN; pyoluteorin, PLT; non-ribosomal peptide synthase, NRPS; ^T^ means type strain.

**Table 4:** The minimum inhibitory concentration (MIC) and minimum bactericidal concentration (MBC) of DAPG, MAPG, PG, and vancomycin on *S.* *aureus* strains.

Strains	MIC (μg/mL)				MBC (μg/mL)			
	DAPG	MAPG	PG	Vancomycin	DAPG	MAPG	PG	Vancomycin
*S. aureus* ATCC25293	4	>128	>128	2	32	>128	>128	32
*S. aureus* ATCC29213	4	>128	>128	2	32	>128	>128	16
*S. aureus* Sta403	4	>128	>128	2	16	>128	>128	16
*S. aureus* Sta24-1	4	>128	>128	1	32	>128	>128	32

## Data Availability

The accession numbers of the 16S rRNA gene sequence and the draft genome sequence in GenBank are OL377853 and JAJQXB000000000, respectively.

## Supplementary Materials

The following supporting information can be downloaded at: https://www.mdpi.com/article/10.3390/microorganisms10020315/s1, Figure S1: (A) Anti-MRSA activity of strains isolated from rhizosphere. (B) Antibacterial activity of strain FJKB0103; Figure S2: (A) Neighbour-joining phylogenetic tree based on 16S rRNA gene sequences showing the relationship of strain FJKB0103 to the closely related strains. (B) Neighbour-joining phylogenetic tree based on MLSA (16S rRNA, *gyrB*, *rpoD* and *rpoB*) showing the relationship of strain FJKB0103 to the closely related strains; Figure S3: The growth curves of *S. aureus* Sta24-1 with DAPG at different concentrations; Figure S4: Antifungal activity of *P. protegens* FJKB0103 and its mutants against *Rhizoctonia solani*; Figure S5: The swarming motility of *P. protegens* FJKB0103 and its mutants on soft (0.3% *w*/*v*) LB agar plates; Table S1: Strains and plasmids used in this study; Table S2: Primers used in this study; Table S3: Biochemical characteristics of strain *P. protegens* FJKB0103 and *P. protegens* CHA0 ^T^; Table S4: Secondary metabolite and antibiotic gene clusters in *P. protegens* FJKB0103 predicted by antiSMASH.
