# Supplementary material for: Pseudomonas protegens FJKB0103 Isolated from Rhizosphere Exhibits Anti-Methicillin-Resistant Staphylococcus aureus Activity
Source: Microorganisms. 2022 Jan 28;10(2):315. doi: 10.3390/microorganisms10020315 (PMC8877278; doi:10.3390/microorganisms10020315)
Supplement: Supplementary file 1 [file microorganisms-10-00315-s001.zip › microorganisms-1549711-supplementary.pdf]

Pseudomonas protegens FJKB0103 Isolated from Rhizosphere Exhibits Anti-Methicillin-Resistant *Staphylococcus aureus* Activity

Hui Zhao [1, #], Lu Liu [1, #], Lingshuang Yang [1], Qihui Gu [1], Ying Li [1], Jumei Zhang [1], Shi Wu [1], Moutong Chen [1], Xinqiang Xie [1,*], Qingping Wu [1,*]

[1] Guangdong Provincial Key Laboratory of Microbial Safety and Health, State Key Laboratory of Applied Microbiology Southern China, Institute of Microbiology, Guangdong Academy of Sciences, Guangzhou 510070, China; zhaohuichinese@163.com (H.Z.); luckyliulululu@163.com (L.L); yangls8272@163.com (L.Y); guqh888@163.com (Q.G); liying@gdim.cn (Y.L.); zhangjm926@126.com (J.Z.);woshixinqiang@126.com; cmtoon@hotmail.com (M.C.);

# Hui Zhao and Lu Liu contributed equally to this work

* Correspondence: wuqp203@163.com (Q.W.); woshixinqiang@126.com (X.X.)

Supplementary Materials

TABLE S1 | Strains and plasmids used in this study

Strains or Plasmids	Description	References or sources
Strains		
Pseudomonas protegens FJKB0103	Wild-type, isolated from the rhizosphere of *Garcinia mangostana*, Ap^r	This study
ΔphlD	*phlD* gene in-frame deletion in strain FJKB0103, Ap^r	This study
ΔprnA	*prnA* gene in-frame deletion in strain FJKB0103, Ap^r	This study
ΔpltA	*pltA* gene in-frame deletion in strain FJKB0103, Ap^r	This study
ΔorfA	*orfA* gene in-frame deletion in strain FJKB0103, Ap^r	This study
ΔphlD-C	ΔphlD strain complemented with DAPG gene cluster *phlACBD*, Ap^r and Tc^r	This study
Escherichia coli DH5α	φ80 *lacZ*ΔM15Δ(*lacZYA-argF*)*U169 hsdR17 recA1 endA1 thi-1*	Laboratory stock
Staphylococcus aureus Sta24-1	Multi-drug resistant and methicillin-resistant, isolated from food	Laboratory stock
Staphylococcus aureus ATCC25923	Clinical isolate and methicillin sensitive	[1]
Listeria monocytogenes 2568-1	Multi-drug resistant pathogen, isolated from food	Laboratory stock
Bacillus cereus ATCC14579	Food-borne pathogen	[2]
Plasmids		
p2P24	Suicide vector for generation of gene in-frame deletions, *sacB*, Km^r	[16] Yan et al., 2017
p2P24-phlD	Suicide plasmid p2P24 containing deleted *phlD* gene cluster, Km^r	This study
p2P24-prnA	Suicide plasmid p2P24 containing deleted *prnA* gene cluster, Km^r	This study
p2P24-pltA	Suicide plasmid p2P24 containing deleted *pltA* gene cluster, Km^r	This study
p2P24-orfA	Suicide plasmid p2P24 containing deleted *orfA* gene cluster, Km^r	This study
pRK415	*Escherichia-Pseudomonas* shuttle vector, Tc^r	[4] Keen et al. 1988
pRK415-phlACBD	Plasmid pRK415 containing *phlACBD* gene cluster, Tc^r	This study

Ap^r, Tc^r, and Km^r indicate resistance to ampicillin, tetracycline and kanamycin, respectively.

References

[1] Treangen,T.J.; Maybank, R.A.; Enke, S.; Friss, M.B.; Diviak, L.D.; Karaolis, D.; Koren, S.; Ondov, B.; Phillippy, A.M.; Bergman, N.H.; Rosovitz, M.J. Complete Genome Sequence of the Quality Control Strain *Staphylococcus aureus* subsp. *aureus* ATCC 25923. *Genome Announcements* 2014. 2(6), e01110-14

[2] Ivanova, N.; Sorokin, A.; Anderson, I.; Galleron, N.; Candelon, B., Kapatral, V.; Bhattacharyya , A.; Reznik , E.; Mikhailova , N.; Lapidus, A.; Mazur, M.;et al. Genome sequence of *Bacillus cereus* and comparative analysis with *Bacillus anthracis*. *Nature* 2003, 423, 87–91.

[3] Yan, X.; Yang, R.; Zhao, R.X.; Han, J.T.;Jia, W.J.;Li, D.Y.;Wang, Y.; Zhang, N.; Wu, Y.; Zhang, L.Q.; He, Y.X. Transcriptional Regulator PhlH Modulates 2,4-Diacetylphloroglucinol Biosynthesis in Response to the Biosynthetic Intermediate and End Product. *Appl. Environ. Microbiol.* 2017. 83(21). doi: 10.1128/AEM.01419-17

[4] Keen, N.T.; Tamaki, S.; Kobayashi, D.; Trollinger, D. Improved Broad-Host-Range Plasmids for DNA Cloning in Gram-negative bacteria. *Gene* 1988, 70 (1),191-197.

TABLE S2 | Primers used in this study

Primers	Sequences	References or sources
FJKB0103-pltA-17047-*Eco*RI	TAgaattcGTCGTCATATAGGCCTGTG	This study
FJKB0103-pltA-17837-*Xba*I	ATtctagaGCTTGCGCTCGTTGAAG	This study
FJKB0103-pltA-18481- *Xba*I	ATtctagaCTTCTCCAGCGGCGTC	This study
FJKB0103-pltA-19284-*Hin*dIII	ATTaagcttCATGCCGACATAGACCG	This study
FJKB0103-phlD-5273-*Eco*RI	TAgaattcGATCCACAGAATGACCACCG	This study
FJKB0103-phlD-6089-*Kpn*I	TAggtaccGGTGTGCACTGCCAGTTC	This study
FJKB0103-phlD-7422-*Kpn*I	TAggtaccCAACATTGCCAGCGTG	This study
FJKB0103-phlD-5273-*Hin*dIII	ATTaagcttTGCTGGTGGGGGTGAAG	This study
FJKB0103-prnA-2170-*Xba*I	ATtctagaCCGTCAGGCTCTGTCAGTG	This study
FJKB0103-prnA-2789-*Hin*dIII	ATTaagcttGCCGAACAAATGGTAGAAG	This study
FJKB0103-prnA-3624-*Hin*dIII	ATTaagcttCGAGATCGTCTACATGTTCG	This study
FJKB0103-prnA-4334-*Kpn*I	TAggtaccCAGGAAGAAACCGATGTC	This study
FJKB0103-orfA-2422-*Eco*RI	TAgaattcCTTTGCCTGGCGTCTGTAG	This study
FJKB0103-orfA-3070-*Kpn*I	TAggtaccCTTGTCCACCAGCAGCTC	This study
FJKB0103-orfA-4503-*Kpn*I	TAggtaccGCGATTCTCAAGGCCG	This study
FJKB0103-orfA-5153-*Hin*dIII	ATTaagcttGGGCCCGTAGTTGTTGATC	This study
27F	GAGAGTTTGATCCTGGCTCAG	[1]
1492R	CTACGGCTACCTTGTTACGA	[1]

References

[1] Zhao, H.; Ma, Y.N.; Wu, X.G*.; Zhang, L.Q.* *Pseudomonas viciae* sp. nov., Isolated from Rhizosphere of Broad Bean. *Int. J. Syst. Evol. Micr.* 2020, 70(9), 5012-5018.

Table S3. Biochemical characteristics of strain *P. protegens* FJKB0103 and *P. protegens* CHA0^T

Carbon sources	FJKB0103	CHA0^T
Glycerol	+	+
L-Arabinose	+	-
D-Ribose	+	-
D-Xylose	+	-
D-Glucose	+	+
D-Fructose	+	+
D-Mannose	+	+
Inositol	+	+
Mannitol	-	+
N-Acetylglucosamine	-	+
Sucrose	+	-
Trehalose	+	+
D-Fucose	+	-
D-Arabinitol	+	-
2-keto-Gluconate	-	+

+, Positive; –, negative;

Table S4. Secondary metabolite and antibiotic gene clusters in *P. protegens* FJKB0103 predicted by antiSMASH[a]

Gene clusters	Type	Most similar known cluster	Similarity [b]
Gene cluster 1	NRPS	Pyoverdin	21%
Gene cluster 2	NAGGN		
Gene cluster 3	NRPS	Lipopeptide 8D1-1/lipopeptide 8D1-2	6%
Gene cluster 4	CDPS		
Gene cluster 5	Bacteriocin		
Gene cluster 6	NRPS	Thiazostatin/watasemycinA/2-hydroxyphenylthiazoline enantiopyochelin/isopyochelin	26%
Gene cluster 7	Other	Pyrrolnitrin	100%
Gene cluster 8	Betalactone	Fengycin	13%
Gene cluster 9	NRPS	Pyoverdin	14%
Gene cluster 10	Bacteriocin		
Gene cluster 11	T3PKS	2,4-diacetylphloroglucinol	100%
Gene cluster 12	Arylpolyene	APE Vf	40%
Gene cluster 13	T1PKS	Pyoluteorin	100%
Gene cluster 14	NRPS	Orfamide A/orfamide C	94%
Gene cluster 15	NRPS	Pyoverdin	20%
Gene cluster 16	NRPS	Cyanopeptin	75%

[a] Clusters identified by antiSMASH 5.0 using the ‘Extra Features’ settings.

[b] The percentage similarity between genes in predicted clusters and the most similar known cluster. A BLAST e-value <1E-05 was used as a significance threshold for genes showing similarity, along with 30% minimal sequence identity, and a shortest BLAST alignment coverage >25% of the sequence. The gene clusters that similarity to the known secondary compounds biosynthetic gene clusters were more than 90% highlighted in gray.

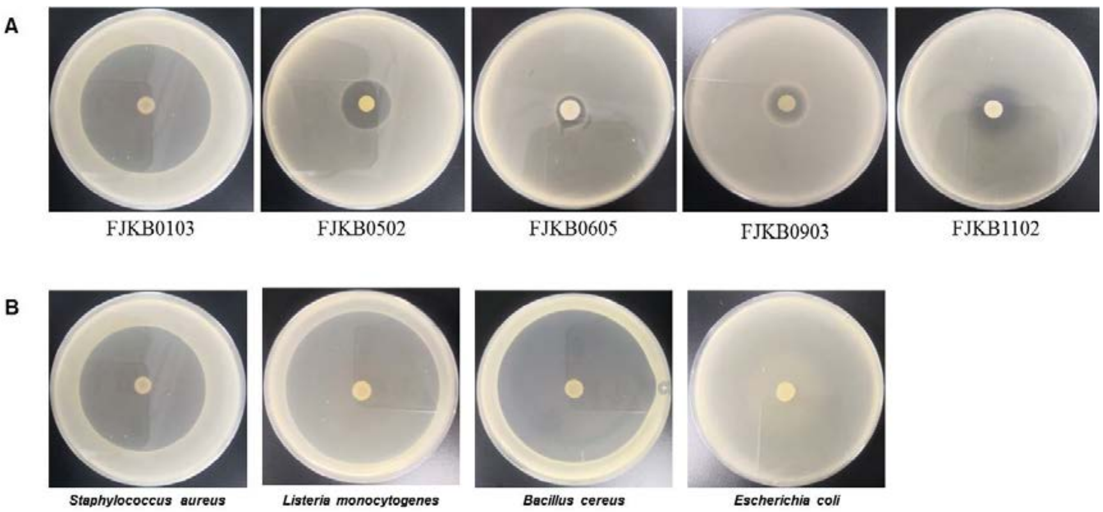

Figure S1. (**A**) Anti-MRSA activity of strains isolated from rhizosphere. (**B**) Antibacterial activity of strain FJKB0103. For antibacterial assays, 5 μL of overnight culture of *P*. *protegens* FJKB0103 was dropped onto the PDA plates center, after inoculating at 28 ℃ for 2 days, the LB mixed with 10% saturated bacteria were poured on the PDA plates, and inoculated at 37 ℃ for 12 h.

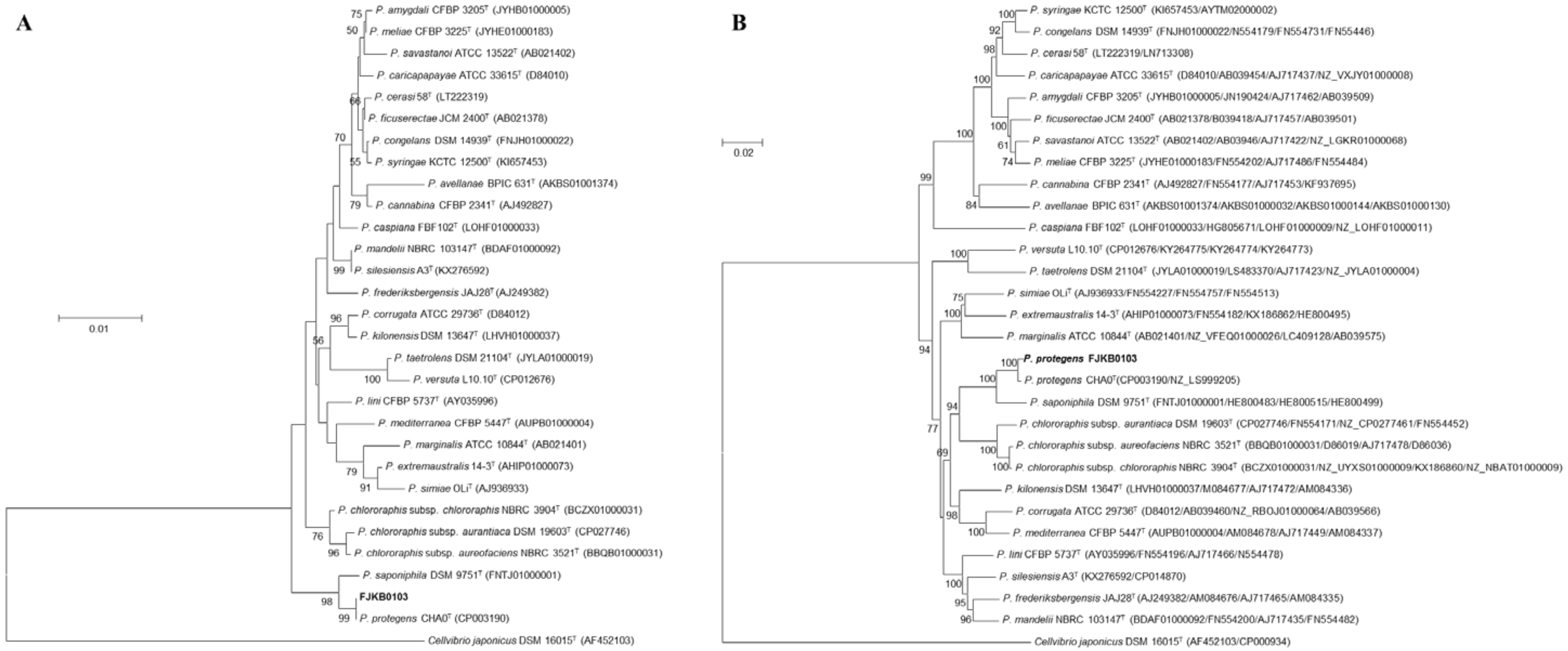

Figure S2. (**A**) Neighbour-joining phylogenetic tree based on 16S rRNA gene sequences showing the relationship of strain FJKB0103 to the closely related strains. GenBank accession numbers of 16S rRNA genes are given in parentheses. (**B**) Neighbour-joining phylogenetic tree based on MLSA (16S rRNA, *gyrB*, *rpoD* and *rpoB*) showing the relationship of strain FJKB0103 to the closely related strains. The sequences of their genes 16S rRNA (1377 bp), *gyrB* (734 bp), *rpoB* (915 bp) and *rpoD* (723 bp) were retrieved from their GenBank depositions or from their whole genome sequences. GenBank accession numbers are given in parentheses (accession numbers are given in parentheses in the following order: 16S rRNA, *gyrB*, *rpoB* and *rpoD* genes. *Cellvibrio japonicas* DSM 16015^{T} was used as an outgroup. Numbers at nodes are bootstrap values (≥50%) from 1000 repetitions.. The strain FJKB0103 was highlighted in bold. T means type strain.

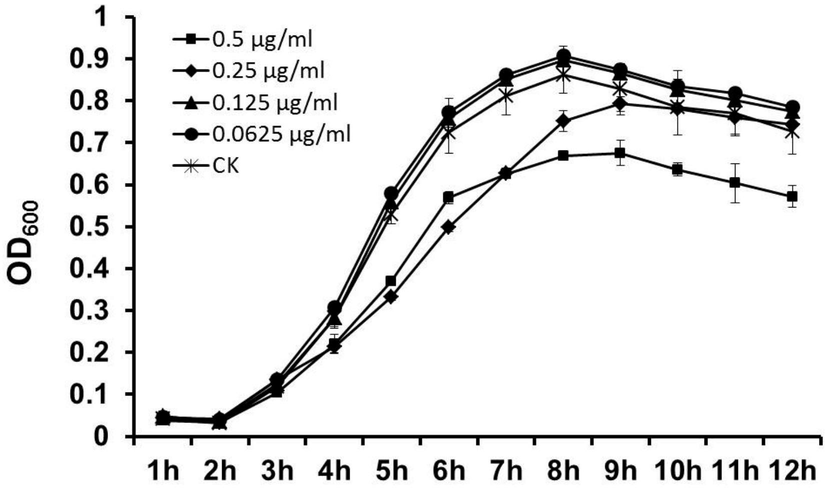

Figure S3. The growth curves of *S. aureus* Sta24-1 with DAPG at different concentrations. The experiment was performed in triplicate, and the mean values ± SD are indicated.

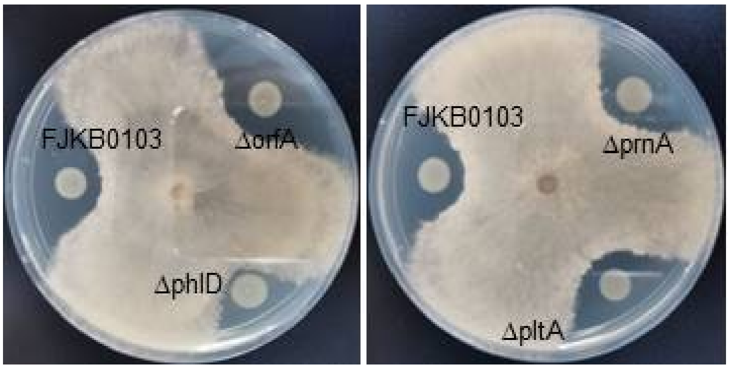

Figure S4. Antifungal activity of *P*. *protegens* FJKB0103 and its mutants against *Rhizoctonia solani*. *R. solani* discs (5 mm diameter) were placed in the center of each plate and 5 μL of an overnight culture of wild-type FJKB0103 or its mutants were inoculated near the edge of PDA medium plates. FJKB0103, the wild-type strain of *P. protegens* FJKB0103; ΔphlD, the *phlD* gene deleted mutant of *P. protegens* FJKB0103; ΔorfA, the *orfA* gene deleted mutant of *P. protegens* FJKB0103; ΔpltA, the *pltA* gene deleted mutant of *P. protegens* FJKB0103; ΔprnA, the *prnA* gene deleted mutant of *P. protegens* FJKB0103.

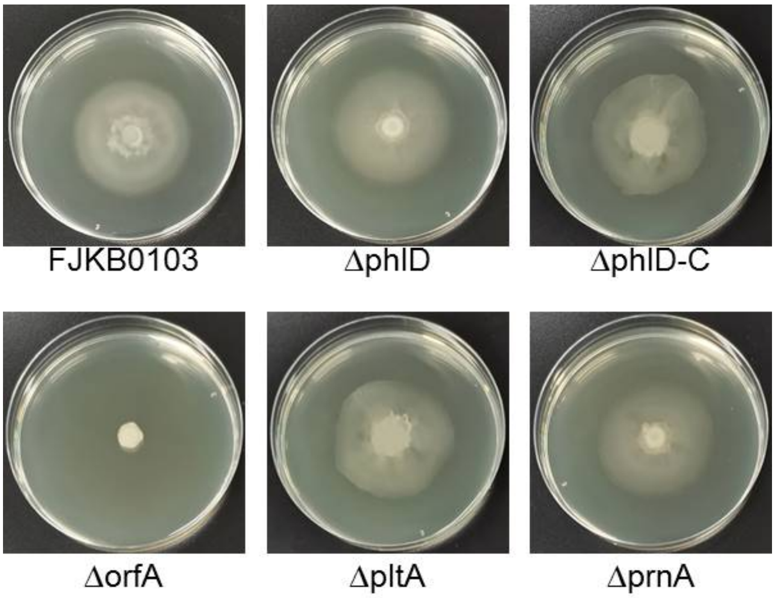

Figure S5. The swarming motility of *P. protegens* FJKB0103 and its mutants on soft (0.3% w/v) LB agar plates. Samples (5 μL) of overnight cultures were spot-inoculated in the center of a soft agar plate and incubated for 24 h at 28°C. FJKB0103, the wild-type strain of *P. protegens* FJKB0103; ΔphlD, the *phlD* gene deleted mutant of *P. protegens* FJKB0103; ΔphlD-C, the complementary strain of ΔphlD with pRK-phlACBD; ΔorfA, the *orfA* gene deleted mutant of *P. protegens* FJKB0103; ΔpltA, the *pltA* gene deleted mutant of *P. protegens* FJKB0103; ΔprnA, the *prnA* gene deleted mutant of *P. protegens* FJKB0103.
